# Identification of the ^15^FRFG domain in HIV-1 Gag p6 essential for Vpr packaging into the virion

**DOI:** 10.1186/1742-4690-1-26

**Published:** 2004-09-13

**Authors:** Henghu Zhu, Heng Jian, Ling-Jun Zhao

**Affiliations:** 1 Institute for Molecular Virology, St. Louis University School of Medicine, St. Louis, USA; 2 Department of Plant Pathology, China Agricultural University, Beijing, China

## Abstract

The auxiliary regulatory protein Vpr of HIV-1 is packaged in the virion through interaction with the Gag C-terminal p6 domain. Virion packaging of Vpr is critical for Vpr to exert functions in the HIV-1 life cycle. Previous studies suggest that Vpr interacts with a (Lxx)4 domain in p6 for virion packaging. In the present study, mutational analysis of HIV-1 Gag p6 domain was performed in the context of the HIV-1 genome to examine the effect on virion packaging of Vpr. Surprisingly, Ala substitutions for Leu^44 ^and Phe^45 ^in the (Lxx)4 domain or deletion of the whole (Lxx)4 domain (amino acid #35–52 of the Gag p6 domain) did not affect Vpr virion packaging. Vpr virion packaging was normal when amino acid #1–23 of the Gag p6 domain was preserved. Most importantly, Ala substitutions for Phe^15^, Arg^16 ^and Phe^17 ^in the context of amino acid #1–23 of the Gag p6 domain abolished Vpr virion packaging. Single Ala substitutions for Phe^15 ^and Phe^17 ^also abolished Vpr virion packaging, whereas Ala substitution for Arg^16 ^had no effect. Our studies have revealed a novel signal sequence for Vpr packaging into the HIV-1 virion. The ^15^FRFG domain in p6 resembles the FxFG repeat sequences commonly found in proteins of the nuclear pore complex. These results have provided novel insights into the process of virion packaging of Vpr and suggest for the first time that Vpr may recognize the FxFG domain for both virion packaging and association with nuclear pores.

## Findings

Vpr is a 15 kDa auxiliary regulatory protein of HIV-1 produced in the late phase of the viral life cycle and packaged in the virion [1-3]. Thus, Vpr has the capacity to function both in the early phase and the late phase of the viral life cycle. A number of biological activities have been assigned to Vpr, including nuclear localization [4-6], transcriptional effects [7,8], cell cycle arrest at the G2/M check point [9-13], and pro- and anti-apoptotic activities [14-18]. In most cases the direct cellular target for Vpr remains to be identified. It is possible that Vpr has multiple unrelated functions to facilitate HIV-1 interaction with the host cells. Alternatively, some of the biological activities of Vpr may be explained by a common mechanism.

Transiently expressed Vpr localizes in the nucleus, and specific nuclear localization signals have been identified in Vpr [6]. Vpr nuclear transport has been correlated with interaction with importin a [19]. However, the nuclear localization of Vpr appears to be more complicated since Vpr is also found to interact with residents of the nuclear pore complex [20]. Notably, Vpr is found to interact with the FG repeat domain of rat Poml21, which is a nuclear pore protein [20]. However, in similar assays Vpr fails to interact with the FG repeat domain of other nuclear pore proteins [20]. Thus, the exact specificity of this interaction remains uncharacterized.

Virion packaging of Vpr is through interaction with the Gag C-terminal p6 domain [1]. With vaccinia expression of HIV-1 Gag and Vpr, a (Lxx)4 domain (amino acid #35–46) in HIV-1 p6 was determined to be essential for virion packaging of Vpr [21]. Fusion of MLV Gag with the HIV-1 p6 domain allows the fusion protein to package Vpr [22]. Under this condition, single point mutations of L45A or F46A within the (Lxx)4 domain abolish Vpr virion packaging [22]. The direct interaction of HIV-1 p6 with Vpr appears to be rather weak, detectible only by using a sensitive in vitro assay [23]. The dissociation constant for the p6-Vpr complex is between 18–75 μM [23]. It is hypothesized that this weak interaction may be enhanced during the process of virion packaging when Gag forms oligomers [23]. Secondary interactions between Vpr and other regions of Gag may also aid virion packaging of Vpr [24]. Interestingly, the HIV-1 p6 also has well-characterized domains for binding cellular sorting factors Tsg101 and AIP1 [25,26]. Whether these interactions influence Vpr virion packaging remains unclear.

In this study, sequences in HIV-1 Gag p6 domain required for Vpr virion packaging was dissected in the context of the HIV-1 genome. Surprisingly, the previously identified (Lxx)4 domain in p6 is shown non-essential for Vpr virion packaging. Instead, a ^15^FRFG domain in HIV-1 Gag p6, 4 amino acid residues downstream of the Tsg101-binding domain, is found critical for Vpr virion packaging. Since FxFG domains also occurs in nuclear pore proteins, the current finding also suggests for the first time that Vpr may recognize the same FxFG domain for both virion packaging and association with nuclear pores. Thus, the FxFG domain appears to be a favorite signal for in vivo recognition by Vpr. We discuss the impact of this finding in the context of the HIV-1 life cycle.

**Figure 1:**
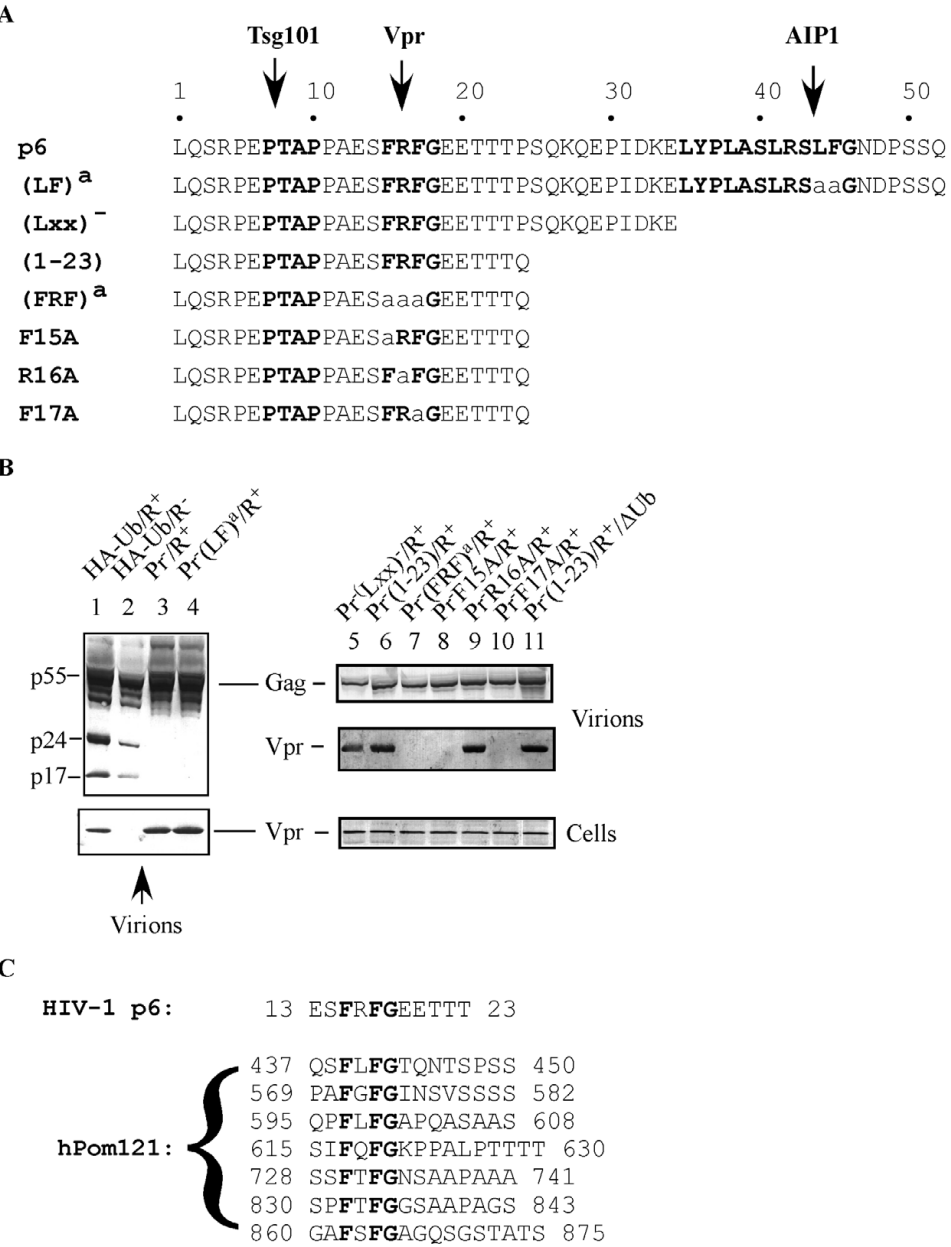
**HIV-1 genomic constructs and requirements for Vpr virion packaging. **A) All viral constructs were based on the p89.6/HA-Ub/R^+^. Pr^-^/R^+^: genomic construct carrying the wild type p6 and a premature stop codon for the protease ORF immediately after the p6 stop codon. All other clones were derived from the Pr^-^/R^+ ^construct. Bold-typed regions represent binding sites for Tsg101, Vpr (this study), and AIP1. B) Effects of p6 mutations on virion packaging of Vpr. Experimental conditions are described in "Findings". Left panels: Gag and Vpr Western blots with virion samples. Right panels: top two panels are Western blots of virion samples, whereas the bottom panel is Western blot of Vpr immunoprecipitated from cell lysates. C) Comparision of the ^15^FxFG domain in HIV-1 Gag p6 with the FxFG domains in human Pom121. HIV-1 p6 sequence is derived from isolate 89.6 [27], and the human Poml21 sequence is derived from GenBank accession number BC008794. Numbers indicate the amino acid positions in the proteins.

To examine the biochemical process of Vpr virion packaging, we introduced various Gag p6 mutations into an HIV-1 genome containing partial deletion of the Pol gene and HA-tagged ubiquitin in place of the Nef gene. This modified HIV-1 genome was used to facilitate construction of p6 mutants and to examine ubiquitination of HIV-1 proteins. All HIV-1 genomic constructs were based on the p89.6 plasmid [27] and their sequences were confirmed by automatic sequence analysis. p89.6/Po1^-^/R^+ ^and p89.6/Pol^-^/R^-^ constructs were described before [28]. A BamHI site was inserted at the beginning of the Nef ORF in a subclone of p89.6 carrying the 3' half of the HIV-1 genome, p89.6/3'[27], to generate p89.6/3'-BamHI. This modification also resulted in deletion of the 5' region of Nef ORF up to the KpnI site. Subsequently, the HA-Ub coding sequence was PCR-amplified from the pCMV-HA-Ub plasmid [29] with primer 1 AGTTACGGATCCATGGCATAGCTACCCTTATGACGTC and primer 2 CATTCAGGATCCTACCCACCTCTGAGACGGAGGACCAG, digested with BamHI and inserted into the p89.6/3'-BamHI plasmid to generate p89.6/3'-HA-Ub. The EcoRI/PstI-blunt fragment of p89.6/3'-HA-Ub was ligated to the EcoRI/SmaI sites of p89.6/Pol^-^/R^+ ^and p89.6/Pol^-^/R^- ^to generate p89.6/HA-Ub/R^+ ^and p89.6/HA-Ub/R^-^constructs, respectively (labeled as HA-Ub/R^+ ^and HA-Ub/R^- ^in Fig. 1).

The p89.6/Pr^-^/R^+ ^and p89.6/Pr^-^(LF)^a^/R^+ ^constructs were prepared by inserting a PstI/StuI digested PCR DNA fragment into the PstI/BalI sites of p89.6/HA-Ub/R^+^. For p89.6/Pr^-^/R^+^, PCR was performed with the p89.6/5' clone as the template [27], and primer 3 GGTACATCAGGCCATCTCACC and primer 4 CTGACCAGGCCTCCCGGGTTATTTTATTGTGACGAGGGGTCGTTGC. For p89.6/Pr^-^(LF)^a^/R^+^, PCR was performed with the same template and primer 3 and primer 5 CTGACCAGGCCTCCCGGGTTATTTTATTGTGACGAGGGGTCGTTGCCTGCGGC TGATCTGAGGGAAGC. For constructs p89.6/Pr (Lxx)^-^/R^+^, p89.6/Pr^-^(1–23)/R^+ ^and p89.6/Pr (FRF)^a^/R^+^, the PCR DNA was digested with PstI/SmaI and ligated into the PstI/SmaI sites of p89.6/Pr^-^ (LF)^a^/R^+^. For p89.6/Pr^- ^(Lxx)^-^/R^+^, PCR was performed with the p89.6/5' template and primer 3 and primer 6 GTACTACCCGGGAGGCCTTTATTCCTTGTCTATCGGCTCCTGC. For p89.6/Pr^-^(l-23)/R^+^, PCR was performed with primer 3 and primer 7 GTACTACCCGGGAGGCCTTTATTGAGTTGTTGTCTCCTCCCCAAACC. For p89.6/Pr^- ^(FRF)^a^/R^+^, PCR was performed with primer 3 and primer 8 GTACTACCCGGGAGGCCTTTATTGAGTTGTTGTCTCCTCCCCGGCCGCGGCGC TCTCTGCTGG. The construct p89.6/Pr^-^F15A/R^+^, p89.6/Pr^-^R15A/R^+^, and p89.6/Pr^-^F17A/R^+ ^were prepared in the same way as p89.6/Pr^-^(1–23)/R^+^, except that the PCR was performed with primer 3 and a new primer instead of primer 7: primer 9 (for p89.6/Pr^-^F15A/R^+^) ACTCGACCCGGGAGGCCTTTATTGAGTTGTTGTCTCCTCCCCAAACCTGGCGC TCTCTGCTGG, primer 10 (for p89.6/Pr^- ^R16A/R^+^) ACTCGACCCGGGAGGCCTTTATTGAGTTGTTGTCTCCTCCCCAAACGCGAAGC TCTCTGC, and primer 11 (for p89.6/Pr^- ^F17A/R^+^) ACTCGACCCGGGAGGCCTTTATTGAGTTGTTGTCTCCTCCCCGGCCCTGAAGC TCTC. The construct p89.6/Pr^- ^(l-23)/R^+^/Δ Ub was prepared by removing the BamHI-BamHI fragment, encoding the HA-tagged Ub gene, from the p89.6/Pr^-^(1–23)/R^+ ^construct.

Cell culture and transfection were performed under conditions described previously [18]. To obtain HIV-1 virions, three days after transfection, culture supernatant was clarified by a low speed centrifugation followed by filtration through a 0.45 nm filter. The clarified culture supernatant was subjected to centrifugation through a 20% sucrose cushion in the SW50.1 rotor at 33,000 rpm for 1 hour. Virions from transfected 293 cells were examined for the presence of Gag and Vpr by Western blot analysis. As shown, Gag p55, p24, p17 as well as Vpr were all detected in the virions with the R^+ ^genome (Fig. 1B, lane 1). With the HIV-1 genome containing a premature stop codon in Vpr (R^- ^genome), no Vpr was detected in the virion (lane 2). We subsequently prepared a protease-truncated construct based on the R^+ ^genome, named Pr^-^/R^+^, and observed normal Vpr virion packaging (Fig. 1B, lane 3). As expected, Gag p55 was not processed with the Pr^-^/R^+ ^construct due to the loss of protease. Surprisingly, normal Vpr virion packaging was still observed with the Pr^- ^(LF)^a^/R^+ ^construct (lane 4), which contains L44A/F45A double mutations in the Gag p6 domain (Fig. 1A) that are reported to abolish Vpr packaging in the context of the MLV Gag/HIV-1 p6 fusion construct [22]. The whole (Lxx)4 domain was then deleted from p6 to generate the Pr^-^(Lxx)^-^/R^+ ^construct, and again normal Vpr packaging was detected (Fig. 1B, lane 5).

The Pr^-^(Lxx)^-^/R^+ ^construct still maintains a ^15^FRFG domain in p6 which resembles the FxFG domain frequently observed in resident proteins of the nuclear pore [30]. To examine the potential involvement of this domain in Vpr packaging, another p6 deletion construct was prepared, with only aa #1–23 of p6 preserved (Fig. 1A). As shown, normal Vpr virion packaging was also observed for this construct, Pr^-^(1–23)/R^+ ^(Fig. 1B, lane 6). Subsequently, ^15^FRF residues were all substituted by Ala residues to generate the Pr^-^(FRF)^a^/R^+ ^construct (Fig. 1A). Importantly, this mutant failed to package Vpr into the virion (Fig. 1B, lane 7).

To examine the roles of individual amino acid residues in the ^15^FRFG domain during Vpr packaging, Phe^15^, Arg^16 ^and Phe^17 ^were individually substituted by Ala (Fig. 1A). As shown, while single F15A and F17A mutations abolished Vpr packaging (Fig. 1B, lanes 8 and 10), R16A mutation had no effect (lane 9). Since all of the HIV-1 constructs expressed HA-tagged ubiquitin (HA-Ub), the HA-Ub coding sequence was removed from the Pr^-^(1–23)/R^+ ^construct. As shown, removal of HA-Ub had no effect on Vpr virion packaging (Fig. 1B, lane 11). Analysis of cell lysates showed that all HIV-1 genomic constructs expressed the same amount of Vpr in the cell (Fig. 1B, lanes 5–11, bottom panel). These results strongly suggest that the ^15^FRFG domain is critical for Vpr virion packaging.

In this report we provide evidence that HIV-1 Vpr is packaged into the virion through the previously unrecognized ^15^FRFG domain in the Gag p6 domain. The Vpr packaging function of the ^15^FRFG domain is preserved when amino acid #1–23 of p6 is retained. This function is abolished when ^15^FRF are substituted by Ala residues. Our conclusion is further supported by the finding that Ala substitutions for Phe^15 ^and Phe^17^abolish Vpr packaging whereas Ala substitution for Arg^16 ^has no effect. Previous studies have shown that a (Lxx)4 repeat domain in Gag p6 is essential for Vpr virion packaging [21,22]. The exact reason for the discrepancy is unclear. However, the previous studies were based on vaccinia expression of Gag and Vpr [21] or on the MLV Gag/HIV-1 p6 fusion constructs [22]. It is possible that different experimental conditions affect the virion packaging of Vpr. Alternatively, different HIV-1 strains may prefer the ^15^FRFG domain or the (Lxx)4 domain for Vpr packaging. It is noticeable that although the ^15^FRFG domain is highly conserved among different HIV-1 strains, it is replaced with ^15^FRSG in the HIV-1 Hxb2 strain (GenBank accession number K03455) and ^15^VRFG in the Yu-2 strain (GenBank accession number AF287352). Future studies may reveal if an engineered FRFG domain in these HIV-1 strains can allow Vpr packaging in the absence of the (Lxx)4 domain.

Significantly, the ^15^FRFG domain of p6 resembles the FxFG domains of certain nucleoporins with respect to both the FxFG core and the following hydrophilic residues rich in Ser/Thr residues (Fig. 1C). Thus, Vpr appears to recognize the same sequence for both virion packaging and association with the nuclear envelope for transport into the nucleus. We hypothesize that the FxFG domain is one of the most important signals for Vpr recognition in vivo. It may govern Vpr function during both the late phase and the early phase of the HIV-1 life cycle.

Vpr interaction with nucleoporins has been reported before [20]. In particular, Vpr is found to interact with the FG repeat domain of Pom121 and more weakly with that of Nsp1p [20]. It has been suggested that the FG residues in these FG repeats constitute the hydrophobic core that is critical for recognition by other proteins [30]. However, the property of this hydrophobic core and the specificity of protein-protein recognition are critically dependent on the neighboring residues preceding the FG residues, so that the FxFG, GLFG, and other types of FG repeats may be involved in different protein-protein interactions [30]. Comparison of the Gag FxFG domain with the seven of the FxFG repeats of the human Pom121 reveals that these FxFG domains are followed by a sequence rich in Ser/Thr residues (Fig. 1C) which may be critical for the function of the FxFG domain. The roles of these Thr residues in Vpr virion packaging remain to be dissected.

It is likely that Vpr recognizes the FxFG domain and not other types of FG repeats. Single Ala substitution for Phe^15 ^in the ^15^FRFG domain of p6 abolishes Vpr virion packaging (Fig. 1). The nucleoporin Nup159p does not interact with Vpr [20], and its FG repeat domain contains eight PxFG repeats and no FxFG repeat. In contrast, the FG repeat domain of the Vpr-interacting nucleoporin Pom121 contains seven copies of the FxFG repeats and six copies of the PxFG repeat. Another nucleoporin that interacts with Vpr weakly, Nsp1p, has a large number of FxFG repeats. However, it is expected that nucleoporins function in the context of a large protein complex and their conformations and interaction with Vpr may be influenced by the presence of other interaction partners.

## List of abbreviations

MLV: murine leukemia virus; Ub: ubiquitin.

## Competing interests

None declared.

## Authors' contributions

HZ and HJ participated in the construction of mutant HIV-1 genomes, cell culture, transfection, and Western blot analyses. LZ conceived of the study and participated in its design, coordination and execution. All authors read and approved the final manuscript.
